# Automatic question answering for multiple stakeholders, the epidemic question answering dataset

**DOI:** 10.1038/s41597-022-01533-w

**Published:** 2022-07-21

**Authors:** Travis R. Goodwin, Dina Demner-Fushman, Kyle Lo, Lucy Lu Wang, Hoa T. Dang, Ian M. Soboroff

**Affiliations:** 1 National Library of Medicine, Bethesda, MD USA; 2 Allen Institute for AI, Seattle, WA USA; 3 National Institute of Standards and Technology, Gaithersburg, MD USA

**Keywords:** Research data, Computer science, Health care, Machine learning, Literature mining

## Abstract

One of the effects of COVID-19 pandemic is a rapidly growing and changing stream of publications to inform clinicians, researchers, policy makers, and patients about the health, socio-economic, and cultural consequences of the pandemic. Managing this information stream manually is not feasible. Automatic Question Answering can quickly bring the most salient points to the user’s attention. Leveraging a collection of scientific articles, government websites, relevant news articles, curated social media posts, and questions asked by researchers, clinicians, and the general public, we developed a dataset to explore automatic Question Answering for multiple stakeholders. Analysis of questions asked by various stakeholders shows that while information needs of experts and the public may overlap, satisfactory answers to these questions often originate from different information sources or benefit from different approaches to answer generation. We believe that this dataset has the potential to support the development of question answering systems not only for epidemic questions, but for other domains with varying expertise such as legal or finance.

## Abstract (summary)

**Table:** 

Measurement(s)	Text
Technology Type(s)	Natural Language

## Background & Summary

The COVID-19 pandemic has highlighted two major problems with access to reliable domain-specific information: (1) information providers are rapidly generating reams of potentially vital information that might never reach their intended audience, and (2) information seekers struggle to find answers to their specific and changing information needs in the rapidly growing and changing body of communications. These two problems are effectively two sides of the same coin, and making information easier to discover reduces the potential for vital information to remain overlooked by its intended audience. Information Retrieval (IR) systems (e.g., search engines such as Google, Bing, or PubMed) are now an integral part of information seeking and one of the main tools used to satisfy information needs. The pandemic exposed a major problem when relying on information retrieval systems under stressful and fast-changing circumstances: existing solutions retrieve hundreds or thousands of documents –- many of which may not be relevant to the user’s specific information need. This needlessly increases the time and mental effort required by information seekers who must read through these documents to find answers to their information needs. Question Answering is a research area aiming to alleviate the problem of finding pertinent information from thousands of documents by bringing the most salient points of relevant documents to focus immediately, saving time and highlighting information that may have been overlooked.

To this end, we propose a new question answering dataset: **Epi**demi**c QA** (EPIC-QA), intended to evaluate automatic approaches for answering ad-hoc questions about the disease COVID-19, its causal virus SARS-CoV-2, related coronaviruses, and the recommended response to the pandemic. While COVID-19 has been an impetus for a large body of emergent scientific research and inquiry, the response to COVID-19 raises questions for the general public. The rapid increase in coronavirus literature and evolving guidelines on community response creates a challenging burden not only for the scientific and medical communities but also the general public to stay up-to-date on the latest developments. Consequently, the goal of the dataset is to evaluate systems on their ability to provide timely and accurate expert-level answers as expected by the scientific and medical communities as well as answers in suitable language for the general public. The EPIC-QA dataset consists of (a) questions collected from various stakeholders who need information on COVID-19, (b) documents – including scientific research articles suited for experts as well as relevant news, social media, and pages from authoritative websites intended for the general public, and (c) answers to these questions extracted from documents in the dataset and systematically annotated and judged by experts.

Only a handful of publicly available health-related QA datasets exist. In terms of information retrieval, CORD-19^1^ is the most widely used document collection, containing documents from PubMed, research articles from the World Health Organization (WHO), pre-prints from bioRxiv, medRxiv, and arXiv, and SemanticScholar. CORD-19, however, does not provide questions or answers. In terms of COVID question answering, CovidQA^2^ provides 124 question–article–answer triplets from 85 articles; COVID-Q^3^ consists of 1,690 questions (without answers) annotated into 15 categories and 207 clusters; Medical Question Pairs (MQP)^4^ contains 3,048 pairs of medical questions annotated as being similar or dissimilar to COVID-19 FAQs; the Synergy Task of the 2021 BioASQ Challenge (http://www.bioasq.org/) included four rounds of binary, factoid, list, and summary questions based on the CORD-19 collection; and the 2021 TREC Health Misinformation Track (https://trec-health-misinfo.github.io/) includes topics focused on COVID-19 with annotations indicating whether documents contradict the topic’s answer. For a detailed review of additional natural language processing resources for COVID-19, see Chen *et al*. (2021)^5^. By contrast, EPIC-QA, which incorporates both public-facing and expert-level documents, is the only dataset exploring the difference between answers suitable for the general public and those appropriate for experts. It is the only dataset designed to include a wide variety of unique possible answers for each question.

**Fig. 1:**
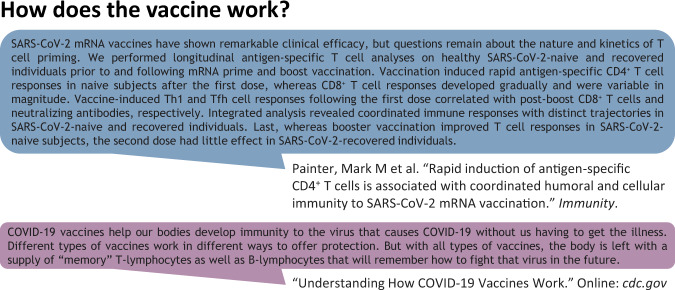
Examples of different answers appropriate for experts and the general public, respectively.

While there is overlap in the types of questions asked by different stakeholders, the answers to such questions should vary based on the background knowledge of the user. For example, consider the simple question illustrated in Fig. 1, *How does the vaccine work?* In the eyes of an expert, an answer should indicate the exact mechanism and pathways involved. By contrast, for a member of the general public, this information is too involved and may cause confusion; instead, a more appropriate answer would provide a more general overview of the role of the vaccine and a high-level description of its mechanism. In the context of the rapidly accelerating knowledge of COVID-19, managing this duality between expert- and general- level information is even more important. It is our hope that the EPIC-QA dataset will stimulate research in automatic question answering not only to support providing high-quality timely information about COVID-19, but also to support the development of generalizable approaches to meeting information needs in the face of varying levels of expertise for other domains, such as general healthcare, finance, or legal.

## Methods

Question answering systems traditionally involve two main steps: an *information retrieval* step, in which relevant documents are retrieved for a given question, and an *answer extraction* step, in which relevant passages or answers are identified within the retrieved documents and extracted as-is or used to synthesize an answer. Recently, *end-to-end* question answering systems have been developed in which (deep) neural networks are trained to jointly identify relevant passages and extract answers from those passages. Consequently, the EPIC-QA dataset includes two collections intended to facilitate research on (a) answer extraction or (b) end-to-end question answering, respectively. The questions in the answer-extraction dataset are adapted from the queries of the TREC-COVID retrieval shared task (https://ir.nist.gov/trec-covid), and are associated with the set of relevancy annotations from that task. For the end-to-end dataset, we introduce a novel set of questions, and provide relevant documents and answer annotations without associated retrieval relevance rankings. These two collections can be used separately or in concert to study the relationship between document retrieval and answer extraction. Both collections consist of three components:

1. Two sets of manually-produced questions, one asked by the general public as well as another asked by experts pertaining to COVID-19;
2. A collection of documents relevant to COVID-19 including published and pre-print biomedical research articles as well as documents obtained from government websites, news, and social media, automatically parsed into sentences; and
3. A diverse list of manually-produced answers for each question as well as a set of relevant document excerpts with manual sentence-level annotations indicating which answers, if any, are evidenced by each sentence.

To facilitate answer-extraction research, the questions in the answer-extraction dataset are additionally associated with a set of relevant documents as might be retrieved by an optimal search engine, while the questions in the end-to-end dataset are not. We detail the process by which each of these components was produced.

### Generating questions

**Fig. 2:**
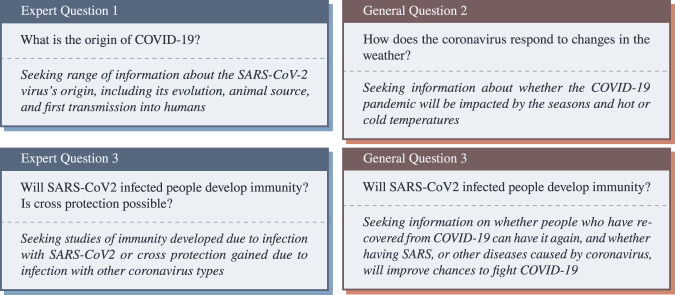
Example questions included in EPIC-QA, shown above their corresponding backgrounds.

Two sets of questions are included: one for expert-level questions and one for the general public. By design, many of the expert and consumer questions overlap in focus to facilitate comparison between both types of stakeholders. For the answer extraction dataset, we adapted the topics evaluated in the fourth round of TREC-COVID (https://ir.nist.gov/covidSubmit). The majority of these questions originated from users’ interactions with MedlinePlus. Additional scientific questions were developed based on group discussions from the National Institutes of Health (NIH) special interest group on COVID-19, questions asked by Oregon Health & Science University clinicians, and responses to a public call for questions. The TREC-COVID topics included expert-level background descriptions of the user’s information need; to facilitate public-facing question answering we produced public-friendly backgrounds for each question. We provide the document-level relevance judgments produced during the TREC-COVID evaluation for these questions. The answer extraction collection includes 45 expert questions and 42 general public questions with document-level relevance judgments. For the end-to-end collections, a new set of 30 expert and 30 general public questions were developed, none of which were evaluated in TREC-COVID; consequently, these questions do not come with document-level relevance judgments. Example questions are provided in Fig. 2. While these may seem like a small number of questions (87 answer extraction questions in total and 60 end-to-end questions in total), each question is associated with multiple answers, and 45–60 passages hand-annotated at the sentence level to indicate which answers, if any, are included in each sentence.

### Collecting documents

Each document in EPIC-QA includes explicitly pre-defined *contexts* (a generalization of paragraphs or sections) and sentence boundaries. To support both levels of expertise, the document collections consists of two parts: scientific and medical research, and public-facing online documents.

#### Scientific and research documents

We adapt the collection of biomedical articles released for the COVID-19 Open Research Dataset Challenge (CORD-19)^6^. The dataset was created by the Allen Institute for AI in partnership with the Chan Zuckerberg Initiative, Georgetown University’s Center for Security and Emerging Technology, Microsoft Research, and the National Library of Medicine – National Institutes of Health, in coordination with The White House Office of Science and Technology Policy. The CORD-19 collection includes a subset of articles in PubMed Central (PMC) as well as pre-prints from bioRxiv and medRxiv. Contexts in this collection correspond to automatically identified paragraphs in the articles’ abstracts, or main texts. We include two snapshots of CORD-19: a snapshot from June 19, 2020 to be used with the answer extraction collection, and a snapshot from October 22, 2020, to be used for the end-to-end collection.

#### Public-facing documents

**Fig. 3:**
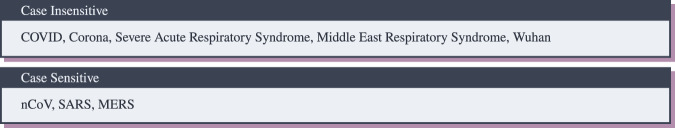
Terms used to test documents for COVID-19 (note: while terms like Corona or Wuhan may appear under-specified, all documents originate from healthcare-focused collections resulting in few false positive matches).

We include a subset of the articles used by the Consumer Health Information Question Answering (CHIQA) service of the U.S. National Library of Medicine (NLM)^7^. This collection includes authoritative articles from the Centers for Disease Control and Prevention (CDC); the Genetic and Rare Disease Information Center (GARD); the Genetics Home Reference (GHR); Medline Plus; the National Institute of Allergy and Infectious Diseases (NIAID); and the World Health Organization (WHO). Contexts in this collection correspond to paragraphs or sections as indicated by the HTML markup of the document. All articles were filtered for COVID-19 content using the terms in Fig. 3. In the end-to-end collection, we also included 265 Reddit threads as well as a subset of the CommonCrawl News Crawl (CCNC) from January 1 through April 30, 2020, as used in the TREC Health Misinformation Track (https://trec-health-misinfo.github.io/). To avoid misinformation, we only considered Reddit threats from the heavily-moderated /r/askscience community that were tagged as pertaining to COVID-19, Medicine, Biology, or the Human Body (and which also contain the COVID-19 terms in Fig. 3). Likewise, to avoid irrelevant pages and misinformation, CCNC documents were filtered by top-level domain (i.e., ".gov" or ".edu"), using the top 100 first-level domains as measured by SALSA^8^, PageRank^9^, and HITS^10^; remaining pages were filtered for COVID-19 content using the terms in Fig. 3.

### Producing answers

When producing answers for the EPIC-QA dataset, our intent was to explore the landscape of answers asserted in the document collection. Thus, we consider any statement that answers the question as an “answer” regardless of whether or not the answer is factually accurate at the time the document was authored or given current information. The answers in this dataset are intended as an intermediary step where-in one would like to explore all answers provided by the document collection – both correct answers as well as incorrect answers that people may have discovered on their own. Answers were produced by applying multiple automatic question answering systems to the questions in EPIC-QA to identify contexts that may answer the question (see *Technical Validation* for more details). These contexts were pooled at a depth of five for expert questions and a depth of eight for questions from the general public, resulting in roughly 45–60 contexts per question. Human assessors read these retrieved contexts and judged them. Specifically, 17 medical indexers with five to thirty years of experience at the National Library of Medicine analyzed the pooled contexts in two rounds: (1) an answer-key generation round, followed by (2) a sentence-level answer annotation round. Annotations were performed as part of the medical indexers’ official duties at the National Library of Medicine.

#### Answer key generation

**Fig. 4:**
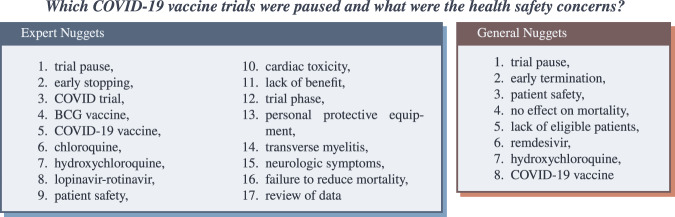
Examples of information “nuggets” or answers produced during answer key generation.

In the first round, assessors had access to a pool of contexts identified for a given question as well as an ad-hoc search engine over the document collection. The role of the answer key generation round was for assessors to explore the contexts identified by automatic question answering systems as well as the document collection to determine a set of atomic “facts” that answer the question based on the question’s information need or background (see Fig. 2). As in Voorhees (2005)^11^, we refer to these facts as *nuggets*, such that each nugget indicates a fact for which the assessor could make a binary decision as to whether a context or sentence contained that fact. Example nuggets that were produced for the question “Which COVID-19 vaccine trials were paused and what were the health safety concerns?” are illustrated in Fig. 4: *lack of benefit*, *patient safety*, etc. The primary role of this round is to create an answer key for the question comprised of nuggets identified from pooled contexts or the assessor’s own ad-hoc search of the collection. The search engine was provided to help assessors explore the topic and identify nuggets that the assessor may be aware of from their prior knowledge of the question but which may not have been included in any of the pooled contexts. Assessors were not expected to exhaustively identify every possible nugget; rather the intent was for them to identify important (at the discretion of the assessor) nuggets that they feel should be included in the answer key based on their understanding of the question.

#### Sentence-level answer annotation

In the second round, the answer key (list of nuggets) was fixed. Assessors were given the same set of pooled contexts used in round one. This time, they were asked to annotate all sentences in each context indicating which nugget(s) (if any) are addressed by each sentence. For example, for the question “Which COVID-19 vaccine trials were paused and what were the health safety concerns?” the sentence “[I]n the light of a recent publication...on the lack of safety and efficacy of HCQ in the treatment for COVID-19 patients, the Executive Group of the Solidarity Trial decided to implement a temporary pause of the HCQ arm within the trial as a precaution, while the safety data is being reviewed” was annotated as containing the expert nuggets: *COVID trial*, *trial pause*, *lack of benefit*, *hydroxychloroquine*, *patient safety*, and *review of the data*.

## Data Records

**Fig. 5:**
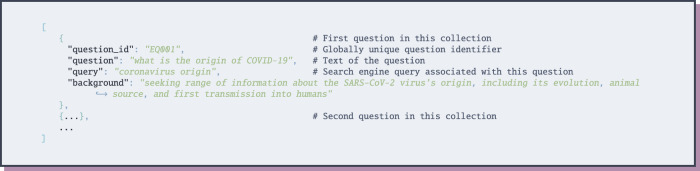
JSON Schema for questions.

The Epidemic Question Answering (EPIC-QA) dataset is available through the Open Science Foundation (OSF) at 10.17605/OSF.IO/VNYK8^12^. The questions in the EPIC-QA dataset are provided in four files following the JSON format illustrated in Fig. 5:

- ae/expert_questions.json contains 45 expert-level questions produced for the answer extraction collection; and
- ae/general_questions.json contains 42 questions from the general public produced for the answer extraction collection.
- e2e/expert_questions.json contains 30 expert-level questions produced for the end-to-end collection; and
- e2e/general_questions.json contains 30 questions from the general public produced for the end-to-end collection.

**Fig. 6:**
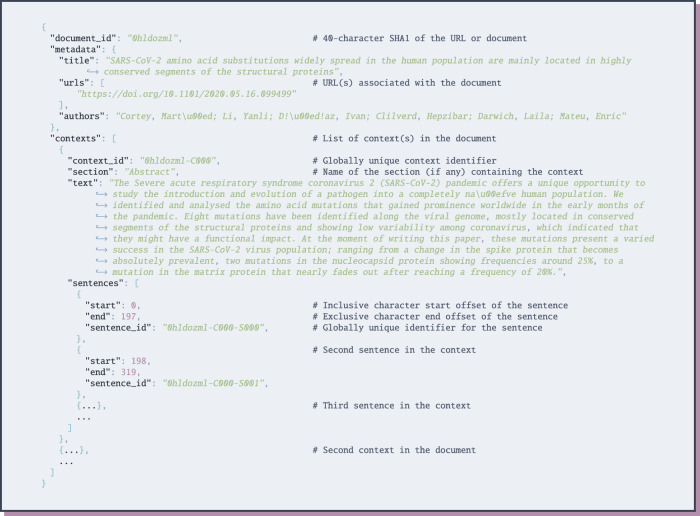
JSON Schema for full-text documents. Note: the full-text of the document is provided within the list of contexts of that document.

The documents included in the EPIC-QA dataset are provided separately for the answer extraction and end-to-end collections. For each collection, each document is provided as a separate JSON file, adhering to the JSON schema illustrated in Fig. 6:

- ae/cord19/ contains 129,069 JSON files, each corresponding to a biomedical research article from a June 19, 2020 snapshot of CORD-19; and
- ae/general/ contains 925 JSON files, each corresponding to a government or health agency website crawled on June 10, 2020.
- e2e/cord19/ contains 236,035 JSON files, each corresponding to a biomedical research article from an October 22, 2020 snapshot of CORD-19;
- e2e/ask_science/ is initially empty (users retain copyright on each of their comments and posts on Reddit, preventing us from redistributing their data directly), however, it can be populated using the Python script scripts/populate_reddit.py to create 263 JSON files, each corresponding to a post made between December 01 2019 and October 29, 2020 to the /r/askscience community on Reddit tagged by community moderators as corresponding to COVID-19, health, medicine, or biology, which contained one of the COVID-19 keywords shown in Fig. 3
- e2e/ccns_trec/ contains 114,645 JSON files, each corresponding to an HTML-parsed website included in the CommonCrawl News Subset used by the TREC Misinformation Track containing one of the COVID-19 keywords shown in Fig. 3; and
- e2e/chqa contains 2,739 JSON files, each corresponding to an October 9, 2020 HTML-parsed snapshot of a web page affiliated with the NIH, CDC, or WHO containing one of the COVID-19 keywords shown in Fig. 3.

**Fig. 7:**
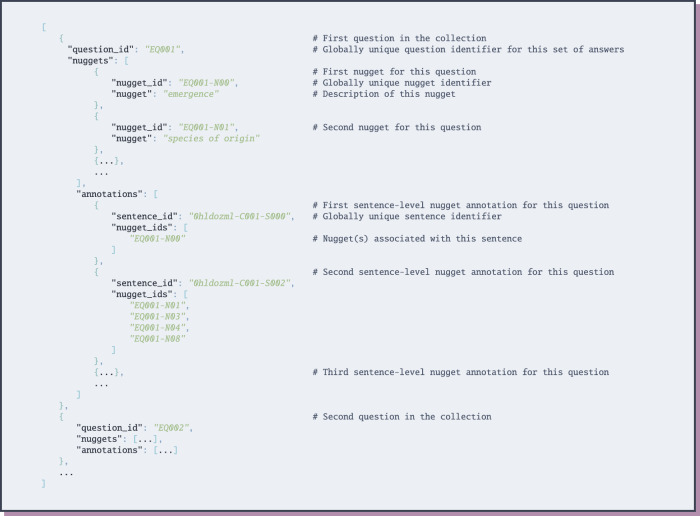
JSON Schema for answers.

**Fig. 8:**
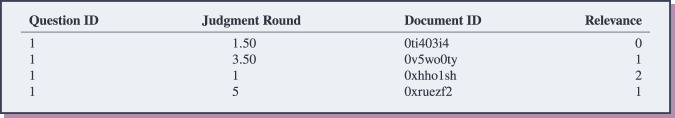
Example format for document-level relevance judgments provided for the answer extraction collection. Question ID indicates which question the relevance judgment was made for; Judgment Round refers to the round of the TREC-COVID evaluation in which the judgment was made (this may be safely ignored for EPIC-QA); Document ID indicates which document was judged; and Relevance indicates the relevance of that document, with 0 for not relevant, 1 for partially relevant, and 2 for fully relevant.

Finally, answers are provided in four files, corresponding to the answer extraction and end-to-end collections of expert and public questions. For each question: each answer (i.e., nugget) is associated with the set of sentence-IDs that nugget was identified within, as shown by the JSON schema in Fig. 7. The answer extraction collection additionally includes document-level relevance judgments in trec_eval (https://github.com/usnistgov/trec_eval) format, as illustrated in Fig. 8. Note: due to time and budget restrictions, only 21 expert and 17 general public questions were fully-annotated with answers in the answer extraction collection. Finally, EPIC-QA also includes the rankings and answers provided by all participants of the associated EPIC-QA evaluation at the Text Analysis Conference (TAC, see *Technical Validation* for details), as well as an “ideal” ranking of answers based on our assessor’s answer annotations.

## Technical Validation

To validate the EPIC-QA dataset, we organized a community evaluation at the 2020 Text Analysis Conference (TAC), in which participants were provided with the dataset (without answers) and asked to submit ranked lists of up to 1,000 relevant *passages* for each question, where a passage was defined as a contiguous sequence of sentences from a single context. We first evaluated the answer extraction collection, and provided the judgments produced from that evaluation as additional data for participants of the later end-to-end evaluation.

We encouraged teams to explore diversity in their answers rather than returning many passages providing the most common or obvious answer. To this end, we validated EPIC-QA using a modified version of Normalized Discounted Cumulative Gain^13^ (NDCG) which we refer to as the Normalized Discount Novelty Score (NDNS). Importantly, while the cumulative gain in NDCG can be computed for a document independently of the other retrieved documents, the Novelty Score (NS) measures the information in a passage that has not been seen previously in the ranked list of passages. Formally, we define the novelty score, NS, of passage *p* as:

$$\begin{array}{lll}NS(p)=\frac{{n}_{a}\cdot ({n}_{a}+1)}{{n}_{a}+{n}_{s}} & DNS({p}_{1},\cdots ,{p}_{l})=\mathop{\sum }\limits_{r=1}^{l}\frac{NS({p}_{r})}{{{\rm{\log }}}_{2}(r+1)} & NDNS({\boldsymbol{p}})=\frac{DNS({\boldsymbol{p}})}{DNS(\widehat{{\boldsymbol{p}}})}\end{array}$$

where ${n}_{a}$ is the number of *novel* answers in passage *p* and ${n}_{s}$ is the number of sentences in the passage (where an answer is considered novel if it was not included in any earlier-retrieved passages for the question.). This metric ensures that identified passages must be brief (i.e., they must express a novel nugget in as few sentences as possible) and they should not contain sentences with only nuggets provided in previous answers. As in NDCG, we (A) compute the discounted cumulative novelty score, DNS, by adjusting the novelty score, NS, of each answer retrieved up to rank $l$ using a logarithmic reduction factor, and (b) normalize the DNS of ranking ${\boldsymbol{a}}={a}_{1},\cdots ,{a}_{l}$ by the DNS of the optimal or ideal ranking of possible answers, $\widehat{{\boldsymbol{a}}}$ that could have been retrieved for that question (based on all the relevance judgments produced for that question). In our evaluation, we used beam-search with a width of 10 to determine the ideal ranking of answers. We report three variations of NDNS which differ in how sentences are counted when calculating $NS$:

1. *Exact*, which prioritizes passages that contain as many novel nuggets in as few sentences as possible, such that ${n}_{s}$ is exactly equal to the number of sentences in the passage;
2. *Partial*, in which the number of sentences used to express novel nuggets is not considered, i.e., ${n}_{s}$ is the number of sentences if all sentences with novel nuggets were merged into a single sentence before counting; and
3. *Relaxed*, in which the number of sentences used to express novel or previously seen nuggets is not considered, i.e., ${n}_{s}$ is the number of sentences if all sentences containing novel nuggets were merged into a single sentence, and all sentences containing previously seen nuggets were merged into a separate single sentence before counting.

The evaluation script is included with the dataset.

### Answer Extraction Validation

A total of 17 submissions were received from eight teams for the expert questions, and 10 submissions from six teams for the general public questions, as shown in Table 1. We observed that the ixa and IBM submissions exhibited the best performance for expert questions while the HLTRI submissions obtained the highest performance followed by IBM for the questions from the general public. The ixa runs used a neural language model powered by SciBERT^14^ fine-tuned on the SQuAD 2.0^15^ general domain question answering data; their second and third runs additionally fine-tuned on QuAC^16^, a dialogue-based question answering dataset involving crowd workers and a teacher discussing a passage of text. Interestingly, their third and best-performing run relied on their own information retrieval system rather than the relevance labels produced during TREC-COVID. IBM’s three runs ignored the included relevance labels, using three different ensembles of elastic search with passage retrieval and/or neural re-ranking approaches. Their final runs were a combination of their ensemble information retrieval scores and scores from RoBERTa^17^ fine-tuned on Natural Questions^18^, a general domain question answering dataset derived from Google search queries and Wikipedia passages. The HLTRI runs relied on a BioBERT^19^-based neural passage re-ranker applied to sentence shingles (runs of multiple adjacent sentences) using the TREC-COVID relevance labels. Overall, while the relevance judgments did help with answering questions for the general public, their impact was less clear for expert-level questions, suggesting that documents may be relevant from an information retrieval perspective despite not having explicitly extraditable answers to the question.

**Table 1:** EPIC-QA answer extraction NDNS results for (a) Expert and (b) General Public questions. .

System	(a) Expert			(b) General Public		
	Relaxed	Partial	Exact	Relaxed	Partial	Exact
CORONAWHY						
Run 1	—	—	—	0.059	0.059	0.043
covidbert						
Run 1	0.143	0.142	0.131	0.364	0.364	0.389
Run 2	0.149	0.149	0.165	0.281	0.276	0.257
Dindadiel						
Run 1	0.148	0.144	0.158	—	—	—
HLTRI						
Run 1	0.302	0.295	0.327	0.488	0.482	0.475
IBM						
Run 1	0.294	0.293	0.325	0.398	0.396	0.413
Run 2	0.293	0.293	0.306	0.374	0.372	0.39
Run 3	0.294	0.294	0.327	0.394	0.394	0.409
ixa						
Run 1	0.276	0.277	0.3	—	—	—
Run 2	0.303	0.304	0.338	—	—	—
Run 3	0.305	0.307	0.341	—	—	—
nlm_lhc_qa						
Run 1	0.113	0.111	0.109	0.278	0.272	0.253
Run 2	0.134	0.132	0.131	—	—	—
UPC_USMBA						
Run 1	0.226	0.218	0.215	0.315	0.307	0.302
Run 2	0.204	0.198	0.212	0.307	0.299	0.286
vigicovid						
Run 1	0.191	0.192	0.192	—	—	—
Run 2	0.266	0.267	0.297	—	—	—
Run 3	0.263	0.265	0.289	—	—	—

Note: teams were not required to use the same system when preparing runs for expert and general public questions.

### End-to-End Validation

For the end-to-end questions, a total of 16 submissions were received from seven teams for expert questions, and 12 submissions from five teams were received for questions from the general public, as shown in Table 2. For expert questions, the HLTRI runs generally exhibited the best performance, though Yastil_R’s first run outperformed HLTRI’s first run. The HLTRI runs relied on a BM25-based information retrieval step^20^, followed by a BERT-based^21^ re-ranker, and a final-step inspired by recognizing question entailment^22^. The first run from Yastil_R relied on an information retrieval step informed by BM25, DeepCT^23^, ColBERT^24^, and docTTTTTquery^25^, followed by an answer-extraction step relying on BERT fine-tuned on MS-MARCO^26^. When looking at the general public, the top performance was exhibited by h2loo, whose runs involved a BM25-based retrieval step followed by point-wise and pair-wise re-ranking using T5, and a final sentence-re-ranking step using maximal marginal relevance (MMR) to improve diversity.

**Table 2:** EPIC-QA end-to-end NDNS results for (a) Expert and (b) General Public questions. .

System	(a) Expert			(b) General Public		
	Relaxed	Partial	Exact	Relaxed	Partial	Exact
h2oloo						
Run 1	0.388	0.34	0.341	0.407	0.359	0.361
Run 2	0.39	0.344	0.344	0.414	0.366	0.368
Run 3	0.376	0.337	0.338	0.382	0.338	0.339
HLTRI						
Run 1	0.408	0.359	0.36	0.346	0.304	0.305
Run 2	0.413	0.363	0.364	0.353	0.312	0.313
Run 3	0.421	0.37	0.371	0.363	0.316	0.317
IBM						
Run 1	0.353	0.315	0.315	0.267	0.249	0.245
Run 2	0.367	0.331	0.329	0.282	0.268	0.264
Run 3	0.354	0.328	0.327	0.278	0.268	0.263
nlm_lhc_qa						
Run 1	0.209	0.223	0.219	0.183	0.186	0.184
vigicovid						
Run 1	0.359	0.329	0.329	—	—	—
Run 2	0.374	0.336	0.334	—	—	—
Run 3	0.391	0.345	0.344	—	—	—
UPC_USMBA						
Run 1	0.148	0.126	0.127	0.033	0.03	0.03
Run 2	—	—	—	0.175	0.176	0.172
Yastil_R						
Run 1	0.41	0.361	0.362	—	—	—
Run 2	0.385	0.338	0.339	—	—	—

Note: teams were not required to use the same system when preparing runs for expert and general public questions.

When comparing between the answer extraction and end-to-end validation results, it is clear that teams generally performed better on end-to-end despite end-to-end being a more challenging problem. This is likely explained by the fact that the answer extraction phase of the TAC challenge was designed and promoted as a preliminary data-collection round, while the end-to-end questions were presented as the primary round for evaluation. It is our belief that the higher performance on the end-to-end evaluation suggests that teams spent more of their research effort on that task than on the answer extraction task.

Overall, teams were able to produce satisfactory answers using a variety of approaches based on the EPIC-QA data. Interestingly, the changing in ranking between expert and public performance also suggests that one-size does not fit all, and that systems need to better account for varying stakeholders, such as experts and the general public.

### Answer Validation

**Fig. 9:**
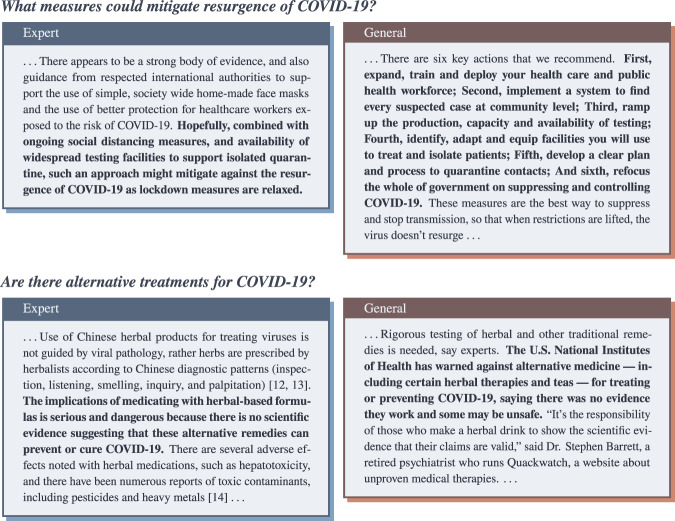
The top answers retrieved by the best-performing automatic systems for two questions in EPIC-QA. The specific answer passage identified by the system is typeset in bold.

Fig. 9 provides the top scoring expert and general answers extracted by the top performing expert (hltri’s third run) and public (h2oloo’s second run) systems developed using the EPIC-QA dataset. Overall, we can see that systems developed using this dataset obtained reasonable results: they were able to identify diverse, concise, and reasonably complete answers that can satisfy experts and the general public. This indicates that the EPIC-QA dataset can be used to train reliable question answering systems to answer epidemic-related questions posed by different stakeholders.

### Impressions

We presented the Epidemic Question Answering (EPIC-QA) dataset, designed to evaluate and draw attention to the important problem of answering questions about COVID-19 from emergent literature, as well as to explore the differences between answers expected by different stakeholders. The EPIC-QA dataset consists of two parts, intended to facilitate answer extraction or end-to-end question answering research, respectively, with each part including (a) two sets of questions collected from two types of stakeholders (experts and the general public), (b) manually-produced answers to these questions extracted from documents in the dataset and systematically hand-annotated and judged by human experts, and (c) a document collection including scientific research articles suited for experts as well as relevant news, social media, and pages from authoritative websites intended for the general public. Technical validation accomplished through a community evaluation at the Text Analysis Conference (TAC) indicates that systems can discover useful answers in the collections, and that answers (and supporting passages) expected by different stakeholders can vary substantially. We believe that our technical validation demonstrates the importance of exploring the diverse landscape of answers available online for health questions and shows the importance of accounting for varying levels of understanding when identifying satisfactory answers to health questions, and hope that this dataset will be of value to researchers exploring answer diversity, multiple stakeholders, or open-ended healthcare question answering.

## Usage Notes

We have provided instructions for how to process this dataset in the README file provided with the dataset. Descriptions of automatic systems developed using this dataset are available at https://tac.nist.gov.

## Data Availability

The code used to prepare the EPIC-QA dataset is provided at https://github.com/h4ste/epic_qa, and a Python script for computing the evaluation metrics reported in the technical validation section of this manuscript is provided with the dataset.
